# Effects of a new approach of aerobic interval training on cardiac autonomic modulation and cardiovascular parameters of metabolic syndrome subjects

**DOI:** 10.20945/2359-3997000000111

**Published:** 2019-03-18

**Authors:** Laís Manata Vanzella, Stephanie Nogueira Linares, Rodolfo Augusto Travagin Miranda, Anne Kastelianne França da Silva, Diego Giuliano Destro Christófaro, Jayme Netto, Luiz Carlos Marques Vanderlei

**Affiliations:** 1 Universidade Estadual Paulista Universidade Estadual Paulista Programa de Pós-Graduação em Fisioterapia Presidente Prudente SP Brasil Programa de Pós-Graduação em Fisioterapia, Universidade Estadual Paulista, Presidente Prudente, SP, Brasil; 2 Universidade Federal de São Carlos Universidade Federal de São Carlos Programa de Pós-Graduação em Fisioterapia São Carlos SP Brasil Programa de Pós-Graduação em Fisioterapia, Universidade Federal de São Carlos, São Carlos, SP, Brasil; 3 Universidade Estadual Paulista Universidade Estadual Paulista Departamento de Educação Física Presidente Prudente São Paulo Brasil Departamento de Educação Física, Universidade Estadual Paulista, Presidente Prudente, São Paulo, Brasil

**Keywords:** Autonomic nervous system, heart rate, blood pressure, metabolic syndrome, syndrome x, exercise

## Abstract

**Objective:**

To evaluate the effects of 16 weeks of periodized aerobic interval training (AIT) on cardiac autonomic modulation and cardiovascular parameters of metabolic syndrome (Mets) individuals.

**Subjects and methods:**

The sample was composed of 52 subjects with a diagnosis of Mets, allocated into two groups: AIT (AITG; n = 26) and control (CG; n = 26). The AITG was submitted to a periodized AIT program, over 16 weeks, while CG was not submitted to any training program. To evaluate the autonomic modulation and cardiovascular parameters in both groups, heart rate variability (HRV) indices, blood pressure (BP), and heart rate (HR) were measured at the beginning and end of the training.

**Results:**

Statistically significant differences were not observed in HFms^2^ (high frequency in milisseconds), LFnu (low frequency in normality unit), HFnu (high frequency in normality unit), and LF/HF ratio indices, or in the cardiovascular parameters BP and HR when comparing the AITG with the CG. However, significant increases in rMSSD (root-means square differences of successive R-R intervals), LFms^2^ (low frequency in milliseconds), and SDNN (standard deviation of normal to normal intervals) were observed in the AITG.

**Conclusion:**

Periodized AIT promoted positive effects on autonomic modulation of Mets subjects, characterized by an increase in the parasympathetic, sympathetic, and global modulation of this population. Additionally, cardiovascular parameter alterations were not observed in Mets subjects submitted to periodized AIT.

## INTRODUCTION

Metabolic syndrome (Mets) is a complex disorder represented by a cluster of at least three cardiovascular risk factors (RF) that include high values of fasting blood glucose, triglycerides, blood pressure, and abdominal circumference and low HDL-cholesterol (1). Mets has a high prevalence around the world, being present in about 23.7% of the American population (2), 30% of the European population (3), 27.4% of subjects in the north of China (4), and 29.6% of Brazilian individuals (5).

Among various alterations promoted by Mets (6–8) are some related to the autonomic nervous system (ANS), characterized by a reduction in vagal and global modulation of ANS and an increase in sympathetic atuation (9). Alterations on autonomic modulation in different conditions like Mets (6–8), can be identified by heart rate variability (HRV) method, a non-invasive technique that describe oscillations between consecutives heart beats (R-R intervals). It is widely used to evaluate ANS influences in sinus node, that can be used to identify physiological stimuli and to disease induced to disorders (10).

Linear methods is one of the ways to analyze HRV. They were divided in time domain analysis and frequency domain analysis. Among the indices in time domain, we can cite: SDNN (standard deviation of normal to normal intervals) and rMSSD (root-means square differences of successive R-R intervals), that represent global and parasympathetic modulation, respectively (10). In frequency domain analysis we can cite the indices: LFms^2^ (low frequency in milliseconds) and LFun (low frequency in normality unit) which is due to the joint action of the vagal and parasympathetic components on the heart, with predominance of the sympathetic ones, HFms^2^ (high frequency in milliseconds), HFun (high frequency in normality unit) which is an indicator of the vagus nerve on the heart, and the LF/HF ratio, that reflects the absolute and relative changes between the sympathetic and parasympathetic components of the ANS (10).

Autonomic alterations described above suggest a physiologic malfunction of the ANS and are related to high vulnerability of the heart and a risk of cardiovascular events (11). In this context, strategies that can act positively on autonomic modulation, such as physical exercise (12–14) should be studied in subjects with Mets to reduce cardiovascular risk in this population. In healthy subjects or those with pathological conditions, aerobic interval training (AIT) has been highlighted for its easy applicability and positive effects that include increases in parasympathetic autonomic modulation and global variability (13,15,16), however, to our knowledge, there are no studies that evaluated the effects of this type of intervention on autonomic behavior in subjects with Mets.

Regarding this type of intervention, in the load dynamics utilized in studies that evaluated these effects on the autonomic modulation of different populations (13,15,16), a lack was observed with respect to the periodization and systematization of the training. Due to its specificity, the existing periodized models of training can be adapted for subjects with pathological conditions and, in this sense, periodization that includes preparatory periods with progressive increases in load, specific phases with less duration of intensity, and periods of transition for recuperation, could be seen as a new type of treatment that promotes security and efficacy in this population.

Taken together, these data point to some gaps in the literature. Could the realization of a periodized AIT program adapted for Mets subjects promote alterations in cardiac autonomic modulation and/or cardiovascular parameters? If so, what will these changes in cardiac autonomic modulation and cardiovascular parameters be?

In this sense, the objective of this study was to evaluate the effects of periodized AIT on cardiac autonomic modulation and cardiovascular parameters in Mets subjects. The hypothesis was that periodized AIT would promote positive alterations in cardiac autonomic modulation and cardiovascular parameters in Mets subjects.

## SUBJECTS AND METHODS

The study is characterized as a nonrandomized control trial, which considers the effects of periodized AIT on cardiac autonomic modulation and cardiovascular parameters in subjects with Mets. The clinical trial was registered in ClinicalTrials.gov (NCT03119493).

The procedures of the study were approved by the Committee for Ethics and Research of the institution (CAAE: 53117116.0.0000.5402). The volunteers were informed about the procedures and aims of this study, and after agreeing to participate, signed a written informed consent form. In addition, each volunteer attached a copy of a medical certificate confirming them to be in a sufficient physical condition to perform the exercises.

### Experimental approach

For realization of this study, an interview was performed with each volunteer to identify personal data (name, age, and sex) and medication in use (investigated throughout the protocol) and all volunteers underwent an initial assessment which included anthropometric measures (height and weight), for sample characterization. After these procedures, blood pressure (BP) was measured. Subsequently, the HR were recorded beat-by-beat using a heart rate monitor (Polar S810i, Finland), for 30 minutes in the supine position, for cardiac autonomic modulation analysis and resting HR acquisition.

After the initial evaluation, the volunteers were allocated by convenience into two groups: periodized AIT group (AITG) and control group (CG) and oriented to maintain their usual diet and daily activities during the study. The AITG were submitted to a 16 week periodized AIT program with a weekly frequency of three sessions, totalizing 48 sessions with a recovery interval between the sessions varying from 24 to 72 hours. The CG realized the same assessments as the AITG, however they did not perform any type of training protocol during the 16 weeks post the initial evaluation. The cardiovascular parameters were assessed and the HR beat-by-beat recorded again in both groups, for analysis of cardiac autonomic modulation, seven days after the end of the training protocol, to minimize possible residual effects of training.

In the AITG, only the subjects that demonstrated at least 85% presence in the periodized AIT protocol were reassessed. Intention to treat analysis was carried out for the remaining volunteers, who, after the period of 16 weeks and final assessments in the control group, were offered the same opportunity to realize the periodized AIT, complying with ethical criteria.

### Population

The study population was recruited through digital media (television), campaigns held in a public square in the commercial center of a city in the interior of São Paulo, and delivery of folders in supermarkets, bank branches, pharmacies, health centers, and the fire brigade.

To define the sample size, the sample calculation based on the rMSSD index obtained in a study of Pichot and cols. (13) was performed. The magnitude of the significant difference assumed was 9 milliseconds (ms), considering a standard deviation of 7 ms, with an alpha risk of 5%, and beta of 80%, the sample size resulted in a minimum of 20 individuals in each group, and this value was increased by 30% considering possible sample losses.

Individuals who had been in regular physical activity in the previous six months, had an inflammatory and/or infectious process, an episode of muscle-tendon or osteoarticular injury in the lower limbs and/or spine, pacemaker, diagnosis of arritmia, had known respiratory, neurological and/or cardiovascular diseases, were excluded from the study.

**Figure 1:**
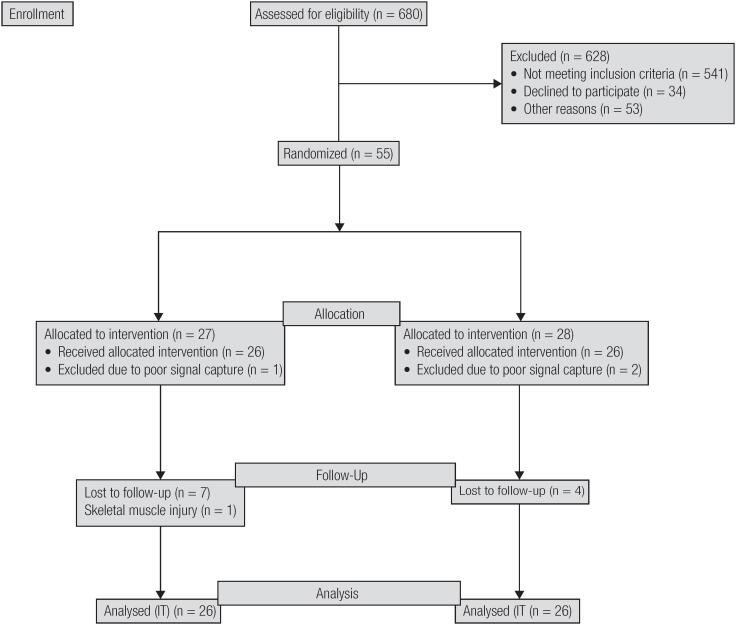
Flow diagram.

Initially, 680 individuals were assessed for eligibility, of which 628 were excluded (541 did not meet the inclusion criteria, 34 gave up participating in the study, and 53 gave up for personal reasons). Fifty-five adult subjects of both genders, aged 40-60 years, and Mets patients according to the criteria defined by the International Diabetes Federation (IDF) (1) participated effectively in the study. In the intervention group, one individual was excluded due to a capitation error, seven dropped out of the study due to transport-related problems to reach the rehabilitation center, and one gave up due to a skeletal muscle injury unrelated to the training protocol. In the control group, two individuals were excluded due to a capture error and four dropped out of the study due to changes in the municipality and personal reasons (Figure 1).

### Training program

The training program was performed on a treadmill (movement, professional LC-160, Brazil and Inbramed, Export, Brazil) for 16 weeks, totalizing 39 sessions, with recovery intervals of between 24 and 72 hours, a weekly frequency of 3 times, and duration of 30 to 75 minutes per session.

Each session of the program was divided into 3 phases: warm-up, training, and cool down. The warm-up had a duration of 10 minutes, with 5 minutes of global stretching of the lower and upper limbs and 5 minutes of walking on the treadmill with a HR less than 20% of heart rate reserve (HRR). The cool down, also lasted 10 minutes and was composed of 5 minutes of walking on the treadmill with a HR of less than 20% of HRR and 5 minutes at rest. To monitor the volunteers, before the warm-up and after the cool down, vital signs were analyzed (BP, HR). Additionally, after the cool down the Borg scale was applied, to verify the perceived effort for each volunteer during the training.

The training phase was executed in a progressive form, with the intensity of training varying between 20% and 90% of HRR, as recommended by the European Association of Cardiovascular Prevention and Rehabilitation, the American Association of Cardiovascular and Pulmonary Rehabilitation, and the Canadian Association of Cardiac Rehabilitation (17). The active recovery between the series was realized at an intensity between 19% and 50% of HRR, according to the intensity of training: mild, moderate, and high.

**Figure 2:**
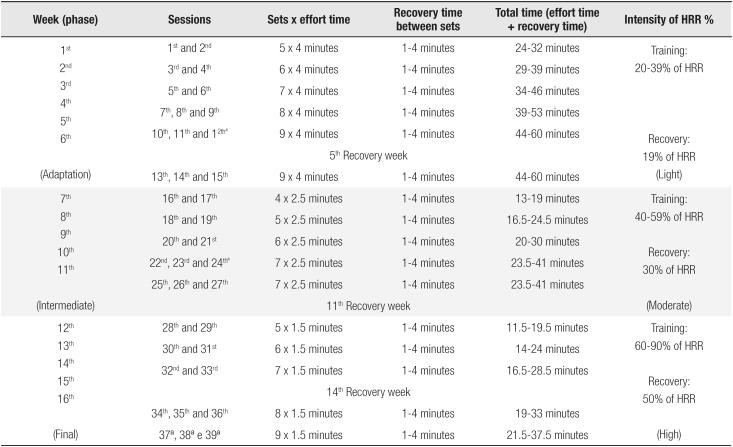
Load dynamics (number of sets and repetitions, effort time, recovery time, total time, and effort intensity) of aerobic interval training.

The load dynamics, that is, the number of series and repetitions (time of effort), time of recovery between the series, total volume of AIT performed, and intensity of the effort are described in Figure 2.

As can be observed in Figure 2, the weeks of training were divided into intensity levels, mild (I) (training range 20-39% of HRR and recovery of 19% of HRR), moderate (II) (training range 40-59% of HRR and recovery of 30% of HRR), and high (III) (training range 60-90% of HRR and recovery of 50% of HRR).

The distributions of load for the AITG, number of series, times of effort were fixed for all volunteers. The time of recovery between the series (1 to 4 minutes), total time (sum of effort total time and recovery time between the series), and speed of effort were variable and established individually considering the subject's response during the session and the percent at which the training was realized. The intensity of effort and HR recovery value were determined though the HRR, a form of prescription recommended by the ACSM (18), which can be easily utilized in clinical practice.

The HRR was obtained by the formula: HRR = (HR_max_ – HR_rest_) x %training + HR_rest_, being the maximal HR (HR_max_) obtained through the Karvonen formula (HR_max_ = 220 – age [in years]) (18,19) and the resting HR (HR_rest_) obtained using a HR monitor with the volunteer at rest for 30 minutes; the average HR at 5 to 25 minutes was obtained using the Polar Pro Trainer program (5.41.002 version).

For the volunteers that used beta blockers, the HR was corrected by the formula: %HR of correction = y + 95.58/9.74 (20) where y is the dose in mg of propranolol or equivalent drug (for the dosage of the equivalent medication of propranolol, the Kaplan table was utilized) (10). The percentage resulting from this formula was subtracted from the HR_max_ for further corrections to the HRR.

During the sessions, the subjects always used the same ergometer and vital signs were monitored (HR and BP), verified at the start of each active recovery period, and signs and symptoms were monitored throughout all sessions.

### Heart rate variability evaluation

Cardiac autonomic modulation was evaluated through heart rate variability (HRV). For this analysis, the HR was recorded beat-by-beat in the morning period (7h to 11h a.m.) in a room artificially heated to between 21ºC and 24ºC with relative air humidity between 40 and 60%. The volunteers were oriented not to consume stimulating substances such as tea, coffee, soda, chocolate, and alcoholic drinks for 24 hour prior to the HRV analysis.

To record HR, a HR monitor (Polar S810i, Finland) was placed on the distal third of the sternum, equipment previously validated for recording heart rate beat-to-beat and calculation of the HRV indices (10).

During the HR recording, the volunteers remained lying on a stretcher at rest, without moving or talking during the execution, and breathing spontaneously for 30 minutes in the supine position. The circulation of people was not permitted in the room during the data collection to reduce the anxiety of the subjects and avoid recording errors.

The data obtained from the HR monitor were transferred to a computer through Polar ProTrainer 5 software (5.41.002 version) and 1000 RR consecutive intervals analyzed, before digital filtering complemented by manual filtering to eliminate ectopic, artifacts, and premature beats, realized by a blind evaluator. Only series with more than 95% sinus beat were included in the study (10). The HRV analysis was performed by linear methods (time and frequency domains) (10).

The rMSSD and SDNN indices were analyzed in the time domain. The rMSSD corresponds to the root mean square of the successive differences between the RR intervals in the record, divided by the number of RR intervals in a given time minus one RR interval. The SDNN corresponds to the standard deviation of all normal RR intervals (10).

In the frequency domain, the spectral components of low frequency (LF: 0.04 – 0.15 Hz) and high frequency (HF: 0.15 – 0.40 Hz) were evaluated, in milliseconds squared (ms^2^) and normalized units (nu), and the relation between low and high frequency components (LF/HF). The spectral analyses were calculated utilizing the algorithm of Fast Fourier transform.

### Cardiovascular parameter

Blood pressure was verified in an indirect manner using a stethoscope (Littman, United State) and aneroid sphygmomanometer (WelchAllyn, Germany) (21). The cardiac frequency was evaluated with a HR monitor (Polar S810i, Finland).

### Data analysis

Descriptive statistics were used to describe the population profile and the results are presented as average, standard deviation, medium, minimum, and maximum, and confidence interval of 95%.

For evaluation of the effects of the training on cardiovascular parameters and cardiac autonomic modulation, the difference between the values obtained at the end and start of the training protocol were compared in both groups. For this and for comparison of the group characteristics, covariance analysis (ANCOVA) was realized. This analysis compared the difference in the average between the control and training group, adjusting for possible confounders, sex, age and high blood pressure (controlled or not by betablockers and Ca+ channel blocker medication), that should be controlled due to their direct relationship with autonomic modulation. The significance level adopted was established at 5% for all tests. The statistical program SPSS (13.0 version) (SPSS Inc., Chicago, IL, EUA) was used for this analysis.

## RESULTS

Table 1 presents the baseline characteristics of the two groups studied as well as the presence of each one of the Mets markers and the medication used by both groups, separated by class. Although it is possible to note statistically significant differences in the variable weight, for the other variables, including BMI, no significant differences were observed.

**Table 1:** Characterization, Mets components, and class of medication used by volunteers separated by groups

	AITG (n = 26)	CG (n = 26)	P value
Age (years)	49.96 ± 6.53 (40.00 – 59.00)	52.44 ± 6.42 (40.00 – 60.00)	0.16
Waist-hip ratio	0.93 ± 0.05 (0.83 – 1.04)	0.91 ± 0.06 (0.72 – 1.04)	0.23
AC (cm)	111.67 ± 10.39 (96.00 – 133.00)	107.61 ± 9.96 (94.50 – 133.00)	0.15
Weight (kg)	95.11 ± 16.39 (64.40 – 127.10)	83.25 ± 16.89 (56.00 – 124.7)	**0.01**
Height (m)	1.71 ± 0.09 (1.52 – 1.91)	1.63 ± 0.08 (1.47 – 1.80)	0.07
BMI (kg/m^2^)	32.38 ± 4.28 (23.94 – 40.56)	30.75 ± 4.42 (23.92 – 39.59)	0.18
**Mets compounded (%)**			
BP	80.76	80.76	
Blood glucose	69.23	69.23	
Triglycerides	73.07	38.46	
Low HDL	57.69	46.15	
WC increased	100.00	100.00	
**Class of medication (%)**			
Ca+ channel blocker	11.53	19.23	
Antagonist of angiotensina II	46.15	34.61	
Thiazide diuretics	30.76	34.61	
Beta blockers	11.23	42.30	
ECA inhibitors	15.38	7.69	
Insulin	7.69	23.07	
Sulfonylurea	3.84	26.92	
DPP4 inhibitor	3.84	3.84	
Metformin	19.23	30.76	
Statin	26.92	30.76	
Fibrate	7.69	0.00	
Thiazolidinedione	0.00	7.69	
Antiplatelet agent	7.69	3.84	
Others	34.61	23.07	

Boldface indicates statistical significance (p < 0.05). Average ± standard deviation (minimum – maximum).

AC: abdominal circumference; BMI: body mass index; Mets: Metabolic syndrome; cm: centimeters; kg: kilogram; m: meters; m^2^: square meters; %: percent; HBP: high blood pressure; HDL: high density lipoprotein; Ca^+^: Calcium; ECA: angiotensin converting enzyme; DPP4: dipeptidyl peptidase-4; HDL: high density lipoprotein.

The effects of periodized AIT on linear indices of HRV in the time and frequency domains and cardiovascular parameters can be visualized in Table 2. An increase in rMSSD, SDNN, and LFms^2^, were observed in the AITG group (p < 0.05). For HFms^2^, HFnu, and LF/HF, no statistically significant differences were observed. For cardiovascular parameters, no significant differences were observed for either group. Despite the absence of significance, the variables DBP and HR reduced in the AITG.

**Table 2:** Comparison of deltas of HRV indices and cardiovascular variables adjusted by sex, age, and medication use to control high blood pressure

Variable	AITG	CG	F	P	Eta squared	Effect size
	Average (SE)	Average (SE)				
rMSSD	6.15 (2.10)	-0.18 (2.14)	4.228	**0.045**	0.084	Moderate
SDNN	8.55 (2.63)	-1.51 (1.68)	6.823	**0.012**	0.129	Moderate
LF (nu)	5.74 (3.29)	-1.67 (3.36)	2.365	0.131	0.049	Low
HF (nu)	-5.72 (3.27)	1.66 (3.34)	2.375	0.130	0.049	Low
LF (ms^2^)	419.84 (123.53)	-7.08 (126.11)	5.560	**0.023**	0.108	Moderate
HF (ms^2^)	109.98 (48.22)	-29.94 (49.23)	3.920	0.054	0.079	Moderate
LF/HF	0.58 (0.63)	-0.109 (0.64)	0.561	0.458	0.012	Low
HR (bpm)	-1.03 (1.81)	0.55 (1.85)	0.360	0.360	0.008	Low
SBP (mmHg)	-1.68 (2.66)	-2.92 (2.71)	0.100	0.754	0.002	Low
DBP (mmHg)	-2.19 (2.10)	0.05 (2.14)	0.533	0.469	0.011	Low

Boldface indicates statistical significance (p < 0.05).

rMSSD: is the root-mean square of differences between adjacent normal RR intervals in a time interval, expressed in milliseconds; SDNN: standard deviation of all normal RR intervals recorded in a time interval, expressed in milliseconds; LF: low frequency; HF: high frequency; SE: standard error; nu: normalized unit; ms: milliseconds square HR: heart rate; SBP: systolic blood pressure; DBP: diastolic blood pressure; bpm: beats per minute; SE: standard error; mmHg: millimeters of mercury.

## DISCUSSION

The results of the present study suggest significant improvement in parasympathetic, sympathetic, and global modulation of HRV in the AITG group, considering the significant increase in rMSSD, LFms^2^, and SDNN indices respectively. For other HRV indices analyzed, including HFms^2^, LFun, HFun, and LF/HF ratio, as well as the cardiovascular parameters SBP, DBP, and HR, statistically significant differences were not observed between the AITG and CG.

Although the effects of the periodized AIT proposed by this study on the autonomic modulation of Mets individuals was not found in the literature (15,22) showed that the utilization of AIT in healthy subjects promoted improvement in parasympathetic and global modulation. Furthermore, beneficial effects on autonomic modulation in individuals submitted to AIT after percutaneous coronary intervention were also found by Munk and cols. after six months of high intensity interval training (23).

In the present study, significant improvements in SDNN and rMSSD indices were observed in the AITG group, suggesting that the periodized AIT performed promoted, respectively, increases in global variability and vagal modulation of the ANS. Furthermore, although not significant, an increase in parasympathetic modulation in the AITG group was also observed in the HFms^2^ index, which presented a moderate effect size.

The mechanisms involved in the increase in HRV, and principally in parasympathetic modulation, in individuals submitted to exercise are speculative. Studies indicate that the fact could be related to a reduction in levels of angiotensin II, a substance that inhibits vagal activity (24). In addition the increase in nitric oxide could be related to an activation of vagal modulation, however further investigations related to this aspect are still necessary (25).

In addition to the indices described above, a significant increase in LFms^2^ was observed in our study. Studies demonstrate that this index is related to the global modulation of ANS with sympathetic predominance (10), furthermore, the LF was related to baroreflex gain, suggesting that it represents the ability to modulate the ANS influence on the heart though the baroreflex action (26).

With regard to baroreflex sensibility, it has been demonstrated that Mets components are related to a cardiovagal baroreflex dysfunction, representing increased cardiovascular risk in this population (27). A significant increase in the LFms^2^ index was observed in individuals of the AITG group, which suggests higher baroreflex gain in this population, and consequently, higher adaptation capacity of the ANS.

With regard to the LF/HF ratio and the LF and HF indices in normalized units, statistically significant differences were not observed between groups. The increase in both indices in the frequency domain (LFms^2^ and HFms^2^) of the individuals that realized the periodized AIT compared to the control group, justifies the absence of significance of the indices expressed above.

The increase in autonomic modulation of the Mets individuals who performed the training protocol suggest a better adaptation capacity to external stimuli and less heart vulnerably to the risk of cardiovascular events (10,11), acting as an important protector mechanism in these individuals.

With respect to the cardiovascular parameters SBP and DBP, Stensvold and cols. (28) and Tjonna and cols. (29) reported that the utilization of high intensity AIT, realized for 12 weeks, was not sufficient to promote significant alterations in this parameter, corroborating with our findings, although both studies presented clinically relevant reductions, since SBP reduced 6 mmHg and 10 mmHg respectively, and DPB reduced 4 mmHg and 5 mmHg respectively. Lower values of SBP (1.68 mmHg) and DBP reduction (2.19 mmHg) were observed in the present study with periodized AIT.

The lower reduction observed could be related to the characteristics of the proposed training. In the model of proposed training, adaptive and intermediate phases were included that preceded the high intensity phase, denominated as the final phase, the latter having a duration of only four weeks. The training models used in the above studies (28,29) utilized high intensity training throughout the period, therefore the intensity of training could influence the lower reduction in the SBP and DBP values found in the present study, when compared to the existing literature (28,29).

In relation to HR, significant differences were not found between the groups studied. Despite this we observed a reduction in these values in the AIT group, possibly attributed to vagal modulation increase, which may not have been sufficient for the HR reduction in these individuals to be considered significant. Other mechanisms may also be responsible for reduction in resting HR such as the effect of aerobic exercise, as in the case of venous return and systolic volume (30).

The realization of the exercise program could increase the venous return and, consequently, blood volume in the cardiac cavity, promoting an increase in myocardial contractility and consequent improvement in systolic volume. In response to an increase in systolic volume, the HR decreases so the cardiac output remains constant (30). This effect is independent of ANS, and the time of periodized AIT or the intensity of the training used may not have been sufficient to promote this effect, which justifies the absence of significance related to this parameter.

The present study presents weaknesses related to the randomization of the volunteers, which was not possible due to the logistics of the training. The proposed training was realized in the evening, which made adherence difficult for individuals that did not have transport and lived far away from the place of the training. These individuals were allocated to the control group, preventing randomization of the volunteers.

Furthermore, a large number of volunteers (80.76%) in TAI and control group had high blood pressure as component of Mets. Several of than were in use of medication that can directly influence in autonomic modulation. To minimize this aspect, the use of medication to high blood pressure were considered as confounding factors and the analyses realized were adjusted by medication use. Moreover, variables like sex and age were also considering as confounding factors during the statistical analysis process. In addition, an intention to treat analysis was realized in individuals that did not complete the periodized AIT. Aspects such as this reinforce our findings.

The present study has an importance clinical implication, since it suggests a new model of training able to produce beneficial effects on the autonomic modulation of Mets individuals, reducing the risk of cardiovascular events in this population. Furthermore, the model of training proposed can be considered safe for Mets individuals since complications related to the cardiovascular system were not observed in the AIT group, and the skeletal muscle injury demonstrated in Figure 1 did not occur during the training.

The absence of cardiovascular and musculoskeletal complications related to the training protocol is an aspect that can be highlighted. We attribute this aspect to the periodization characteristics of AIT, since this consisted of preparatory periods such as progressive increases in load, specific phases with less duration in intensity, and transition periods for recovery, important and positive characteristics of the proposed training.

The period of the protocol realized at an intensity higher than 60% of HHR was short (4 weeks), which may not be sufficient to promote significant responses in cardiorespiratory parameters of individuals in the AIT group. In this sense, the present study suggests that further investigations about the effects of periodized AIT on cardiorespiratory parameters of Mets individuals should be realized, prioritizing a longer duration of the training protocol at intensities higher than 60% of HHR.

We conclude that 16 weeks of periodized AIT suggested positive effects on autonomic modulation of Mets individuals, characterized by an increase in parasympathetic, sympathetic, and global activity in this population. Furthermore, cardiovascular parameter alterations were not evidenced in Mets individuals submitted to the periodized AIT.
